# Lack of validated blood pressure devices for use in pregnancy available from Australian pharmacies

**DOI:** 10.1038/s41440-025-02304-x

**Published:** 2025-08-14

**Authors:** Kaylee Slater, Hollie Speer, Niamh Chapman, Dean S. Picone

**Affiliations:** 1 Sydney School of Health Sciences, Faculty of Medicine and Health, University of Sydney, Sydney, NSW Australia; 2 Research Institute for Sport and Exercise, Faculty of Health, University of Canberra, Canberra, ACT Australia; 3 Menzies Institute for Medical Research, University of Tasmania, Hobart, Tasmania Australia

**Keywords:** Pregnancy, Pharmacy, Blood pressure determination, Implemental hypertension, Digital hypertension

## Abstract

Blood pressure monitoring is a critical aspect of prenatal care, as hypertension during pregnancy can lead to serious complications such as preeclampsia, eclampsia, and other hypertensive disorders. Automatic blood pressure devices are widely used for home monitoring due to their convenience and ease of use. The use of validated automated blood pressure monitors is recommended by the International Society of Hypertension for home blood pressure measurements, and automatic devices require accuracy validation among people who are pregnant before they are recommended for use in pregnancy. This study evaluated availability of such devices from 18 Australian pharmacies. Only four devices (4/54, 7%) were validated for pregnancy and were more expensive than devices validated for the general population (14/54, 26%) and non-validated devices (40/54, 74%). Additionally, limited labelling and information was available to assist consumers to make informed purchasing decisions about home blood pressure devices for use in pregnancy. Increased availability, clear labelling and consumer education could help ensure use of appropriate blood pressure devices in pregnancy.

**Figure:**
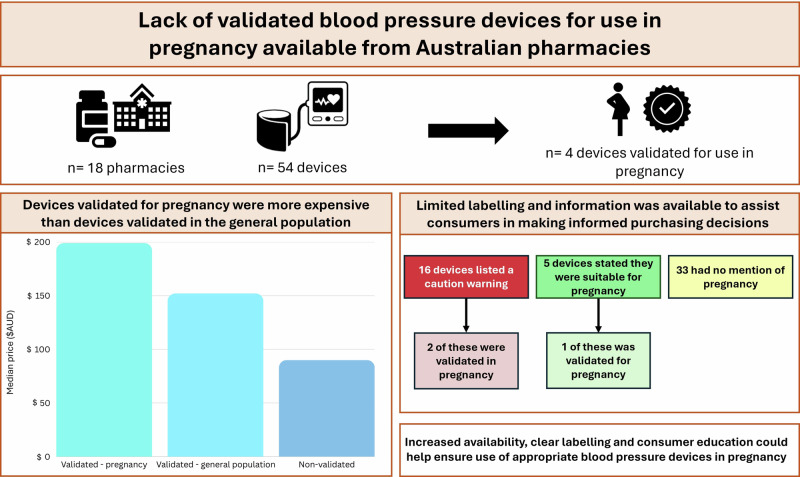
Automatic blood pressure devices require additional accuracy validation for use in pregnancy. We found only four devices (4/54, 7%) were validated for pregnancy, which were more expensive than non-validated devices. Increased education could help ensure use of appropriate blood pressure devices in pregnancy.

Automatic blood pressure devices require additional accuracy validation for use in pregnancy. We found only four devices (4/54, 7%) were validated for pregnancy, which were more expensive than non-validated devices. Increased education could help ensure use of appropriate blood pressure devices in pregnancy.

## Abstract (web summary)

Automatic blood pressure devices require additional accuracy validation for use in pregnancy. We found only four devices (4/54, 7%) were validated for pregnancy, which were more expensive than non-validated devices. Increased education could help ensure use of appropriate blood pressure devices in pregnancy.

## Introduction

Blood pressure (BP) monitoring is a critical aspect of prenatal care, as hypertension during pregnancy can lead to serious complications such as preeclampsia, eclampsia, and other hypertensive disorders [1, 2]. Automatic BP devices are widely used for home monitoring due to their convenience and ease of use [3].

However, only 20% of home BP devices available on the market have been validated for use in general adult populations [4]. To confirm accuracy among pregnant women, automated devices should undergo additional validation testing because of haemodynamic changes induced by pregnancy which may affect the accuracy of the algorithms used by automated oscillometric devices to measure BP [2, 5]. Therefore, even fewer validated devices are available for use in this population [2]. A systematic review of validation studies on office, ambulatory, and home BP measurement devices for pregnant women [6] found that out of 28 devices, 61% passed the validation criteria. However, 66% of these studies were conducted with protocol violations which raises questions about the reliability of the findings [6].

A much larger proportion of BP devices have been validated for general populations than for use during pregnancy. However, the real-world availability of home BP devices that are validated for use during pregnancy is unknown. In Australia, most consumers purchase home BP devices from pharmacies [7], where the availability and validation status of such devices can vary significantly [4]. Therefore, the aim of this study was to determine the availability of home BP devices validated for pregnancy from pharmacies, including device costs and claims regarding use in pregnancy.

## Methods

Pharmacies with physical and online stores in Australia selling upper-arm or wrist cuff BP devices were identified and searched by two independent reviewers (KS/HS) in May-June 2024. The search was conducted on Google Australia using the term “home blood pressure monitor/device”. Validation of devices specifically for use in pregnancy, and the general adult population was determined using the non-profit, hypertension expert-led STRIDE-BP database [8], which provides a rigorously peer-reviewed list of BP devices that have passed accuracy validation testing. Reviewers extracted the device cost and claims about use in pregnancy from instruction manuals and information on pharmacy webpages. Conflicts were adjudicated by other investigators (NC/DP).

## Results

Eighteen pharmacies and 54 unique home BP devices were identified. Four (7%) devices were validated for use in pregnancy and 14 (26%) were validated for use in the general population (Table 1).

**Table 1:** Home BP devices sold in 18 Australian pharmacies

	Number of unique devices found	Validated for use in pregnancy, n (%)	Validated for use in general adult population, n (%)	No evidence of validation in general population or pregnancy, n (%)
**Upper arm cuff**	49	4 (8)	13 (27)	36 (74)
**Wrist cuff**	5	0 (0)	1 (20)	4 (80)
**Total**	54	4 (7)	14 (26)	40 (74)

*BP* blood pressure, The percentages add to more than 100% because some devices validated in the general population were also validated for pregnancy

**Fig. 1:**
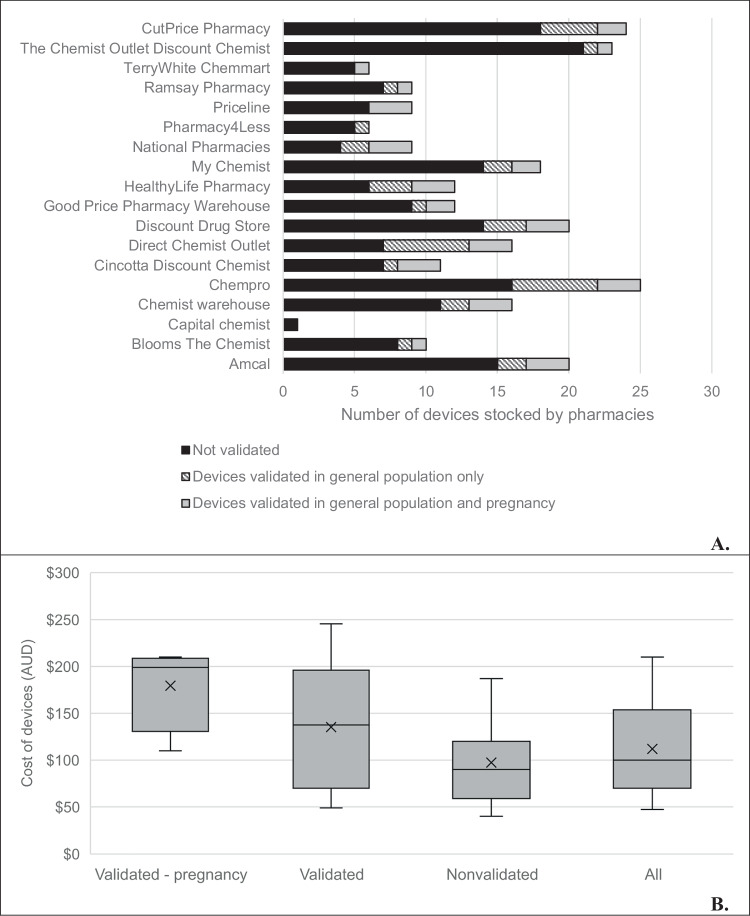
Availability of validated and non-validated devices from 18 Australian pharmacies (**A**, top) and cost of the home BP devices that are validated for use in pregnancy, validated in the general adult population, non validated and all combined (B, bottom). BP: blood pressure. **B** Cost displayed in Australian dollars [14]. Cost could not be determined for one device due to it only being available in-store. Devices labelled ‘validated’ also include those in the validated-pregnancy group. The ‘x’ on the plots represent the mean cost, middle line represents the median cost, the bottom edge of the box is Quartile 1 (value below which 25% of the data points fall) and the top edge is Quartile 3 (75%), the lowest point at the end of the lower whisker represents the lowest cost, and the upper edge of the upper whisker represents the highest cost

Three of the four devices validated for use in pregnancy were assessed against the International Organization for Standardization 81060–2:2018 protocol [9] and the fourth device was assessed against the European Society of Hypertension—International Protocol revision 2010 [10]. A 22–42 cm cuff was used in both studies, which is the size that is sold with all four devices. Sixteen out of 18 pharmacies stocked at least one device that was validated for use in pregnancy (Fig. 1A). The pharmacies operated nationally across a broad geographic and socioeconomic range in Australia. Of the 18 pharmacy chains analysed, five operated stores in every state and territory, and two had a presence in six (out of eight). In this study, pharmacy store coverage was lowest in the Northern Territory (*n* = 5) and Australian Capital Territory (*n* = 7). Six chains had stores in metropolitan, regional and rural areas, and another seven operated in metropolitan and regional areas, while all 18 were present in metropolitan areas.

Devices validated for use in pregnancy were more expensive than those validated in the general population (median $198.99, interquartile range (IQR) 151.47–207.50 versus $137.47, IQR 69.99–192.99). Devices that had no evidence of validation were less expensive ($89.99, IQR 58.95–119.99) (Fig. 1B).

Claims about use in pregnancy were found in either device manuals or pharmacy webpages, and there we no instances where these two sources contradicted each other. Sixteen (30%) of the 54 devices listed a caution warning, suggesting users consult a healthcare provider before using the device in pregnancy. Of these devices, information about four stated they had not been tested among pregnant women and were not suitable for use. This information was only available in device manuals and not at point-of-sale. The manuals from five devices stated a recommended use of the device in pregnancy, although only one of these devices was validated. There was no information identified regarding use in pregnancy for 33 (61%) devices, although one of these devices was validated.

## Discussion

This study reveals a significant gap in the availability of validated BP devices for pregnant women in Australian pharmacies. Out of 54 home BP devices found in Australian pharmacies, only four were specifically validated for use in pregnancy and these devices were substantially more expensive than other devices. Although most pharmacies stocked at least one device that had been validated for use in pregnancy, there was limited information to assist consumers in making informed purchasing decisions at point-of-sale. Improved labelling and stakeholder education is needed to ensure the use of accuracy validated devices during pregnancy in Australia.

A recent systematic review and meta-analysis on the safety and efficacy of home BP monitoring during pregnancy found that home BP monitoring reduced hospital admissions by 70% and lowered the incidence of preeclampsia by 50%, compared to usual care [11]. Nine studies were included in the meta-analysis, however, the authors noted concern about significant heterogeneity and quality of the evidence. Moreover, all the studies were conducted in Europe, United Kingdom and the United States of America, potentially limiting generalisability to other world regions and diverse populations. Indeed, the Society of Obstetric Medicine Australia and New Zealand guidelines state that pregnant women with hypertension might benefit from home BP monitoring with a validated device, but that a current research priority for Australia is more locally relevant data on the utility and safety of home BP monitoring in pregnancy [1].

Despite the potential health benefits of home BP monitoring in pregnancy [12], there is minimal practical guidance for consumers to help them select a validated device, and for healthcare providers when advising patients [2, 4]. A recent real-world study among 127 pregnant women from Australia [12] identified 40 different home BP devices being used by the participants, and although 20% were validated for use in the general population, none were specifically validated for use in pregnancy. In the present study, four devices were marketed as suitable for use in pregnancy despite lacking evidence of clinical validation which is concerning as it may mislead consumers and healthcare providers into relying on potentially inaccurate measurements for clinical decisions. In addition to the minimal practical guidance available to select a device, the substantially higher cost of devices validated for use in pregnancy also has the potential to influence consumer purchasing decisions. The lack of clear labelling and information on the validation status of these devices further complicates the purchasing process, which highlights the need for improved transparency around device validation and increased education and awareness among consumers and healthcare providers about the importance of using validated BP devices during pregnancy. This is especially important for vulnerable populations such as people who are pregnant.

A limitation of this study is that data collection was restricted to Australian pharmacies with physical and online stores. Many online businesses stock home BP devices and previous work from Australia showed only 16% of upper-arm cuff devices from these sources were validated for use in the general population [4]. However, a 2024 mixed-methods study found that the majority of adults who measure their BP at home in Australia purchase their devices through pharmacies [7]. Future research might determine global availability of validated home BP devices for pregnancy, including from e-commerce stores, such as Amazon. Additionally, although there was national presence among the pharmacies, where five chains operated in all territories and states across Australia, we were not able to capture a broad number of pharmacies operating in rural and remote communities as these regions are often serviced by independently owned community pharmacies that may not have online stores. These types of stores did not meet the study inclusion criteria, and the search strategy also did not identify these pharmacies. Therefore, the generalizability of the findings in rural and remote Australia, as well as beyond Australia is unknown. However, previous work on the availability of validated BP devices for the general population has found relatively consistent results between countries [13].

In conclusion, this study has found that there is limited availability of devices validated for use during pregnancy. To promote the use of home BP devices specifically validated for use in pregnancy, it may be necessary to provide clear labelling at point-of-sale. Additionally, education about the need for specific validation of BP devices used during pregnancy may be beneficial for the patients, carers and healthcare providers.

## Data Availability

The data that support the findings of this study are available from the corresponding author upon reasonable request.
